# Natural Enzootic Vectors of *Venezuelan equine encephalitis virus* in the Magdalena Valley, Colombia

**DOI:** 10.3201/eid0901.020136

**Published:** 2003-01

**Authors:** Cristina Ferro, Jorge Boshell, Abelardo C. Moncayo, Marta Gonzalez, Marta L. Ahumada, Wenli Kang, Scott C. Weaver

**Affiliations:** * Instituto Nacional de Salud, Bogota, Colombia; † University of Texas Medical Branch, Galveston, Texas, USA

**Keywords:** encephalitis virus, Venezuelan equine, Culicidae, arthropod vector, arboviruses, alphavirus, research

## Abstract

To characterize the transmission cycle of enzootic *Venezuelan equine encephalitis virus* (VEEV) strains believed to represent an epizootic progenitor, we identified natural vectors in a sylvatic focus in the middle Magdalena Valley of Colombia. Hamster-baited traps were placed into an active forest focus, and mosquitoes collected from each trap in which a hamster became infected were sorted by species and assayed for virus. In 18 cases, a single, initial, high-titered mosquito pool representing the vector species was identified. These vectors included *Culex* (*Melanoconion*) *vomerifer* (11 transmission events), *Cx*. (*Mel*.) *pedroi* (5 transmissions) and *Cx*. (*Mel*.) *adamesi* (2 transmissions). These results extend the number of proven enzootic VEEV vectors to 7, all of which are members of the Spissipes section of the subgenus *Melanoconion*. Our findings contrast with previous studies, which have indicated that a single species usually serves as the principal enzootic VEEV vector at a given location.

Venezuelan equine encephalitis (VEE) is an emerging zoonotic arboviral disease that affects equines and humans in the Americas (*1*). *Venezuelan equine encephalitis virus* (VEEV) has caused sporadic outbreaks since the early part of the 20th century, with some epidemics affecting >100,000 persons. For many years, the source of the epizootic/epidemic VEEV strains belonging to subtypes IAB and IC viruses remained unknown. After antigenically related but distinct, equine-avirulent, enzootic strains of VEEV were isolated in the 1960s, researchers hypothesized that epizootic/epidemic strains evolve from enzootic VEEV progenitors (*2*). The first genetic evidence supporting this hypothesis came from RNA fingerprinting studies that indicated a close relationship between subtype ID–enzootic VEEV strains from Colombia and epizootic/epidemic isolates belonging to subtype IC (*3*). Later, sequencing (*4*) and phylogenetic (*5*,*6*) studies also supported the evolution of the epizootic/epidemic serotype IAB and IC strains from enzootic ID VEEV progenitors. Recently, comprehensive phylogenetic analyses have indicated that the epizootic/epidemic strains evolved independently on at least three occasions from a single lineage of ID VEEV that circulates in eastern and central Colombia, western Venezuela, and northern Peru (*7*–*10*). Other ID-like VEEV lineages that occur in Panama, Amazonian Peru, southwestern Colombia, coastal Ecuador, north-central Venezuela, and Florida have not generated any of the epizootic/epidemic strains sequenced (*10*–*12*).

Enzootic VEEV (subtypes ID–IF, II–VI) circulate nearly continuously in sylvatic or swamp habitats in various tropical and subtropical locations in the New World (*1*,*13*). These viruses generally use small mammals as their reservoir hosts and are transmitted by mosquitoes. Enzootic mosquito vectors have been identified for four VEEV variants: 1) *Culex* (*Melanoconion*) *portesi* transmits *Mucambo virus* (VEE complex subtype IIIA) in Trinidad (*14*), 2) *Cx.* (*Mel.*) *cedecei* transmits *Everglades virus* (VEE complex subtype II) in southern Florida (*15*), 3) *Cx.* (*Mel.*) *aikenii* *sensu lato* (*ocossa* and *panocossa*) transmits subtype ID VEEV in Panama (*16*,*17*), and 4) *Cx.* (*Mel.*) *taeniopus* (formerly *opisthopus*) is the primary enzootic vector of subtype IE VEEV in Guatemala (*18*). More than 70% of enzootic field isolations have come from the subgenus *Melanoconion*, suggesting that these mosquitoes are the principal vectors of most or all enzootic VEE complex strains (*17*).

The infrequency of VEE emergence is probably determined by the infrequent, simultaneous occurrence in time and space of viral mutations that mediate host range changes, combined with ecologic and epidemiologic conditions that permit efficient amplification (*1*). To understand the mechanisms of VEE emergence from enzootic progenitors in Colombia and Venezuela, we are studying the hosts in which epizootic mutations may occur and in which the selection of epizootic strains may follow. However, the vector and reservoir hosts of the particular subtype ID VEEV lineage implicated in epizootic emergence have not been identified. Using an efficient system of vector identification employing hamster baited traps, we identified *Cx*. (*Mel.*) *vomerifer*, *Cx*. (*Mel*.) *pedroi*, and *Cx*. (*Mel*.) *adamesi* as natural enzootic vectors in an active focus of subtype ID VEEV in the middle Magdalena Valley of Colombia.

## Methods

### Study Area

The study was carried out from 1999 to 2000 in the Monte San Miguel Forest in the middle Magdalena Valley of Colombia (6° 23′ 30′′N; 74° 21′ 41′′ W; 50 m elevation). This is a lowland tropical rainforest surrounded by cattle ranches created by deforestation. Mean minimum and maximum daily temperatures are 23°C and 33°C (overall mean of 29°C), respectively, and annual rainfall averages 2,700 mm. Mean relative humidity is 80%. Generally, the peaks of the rainy seasons occur in April–May and October–November. Numerous previous isolations of subtype ID VEEV from sentinel hamsters (*9*) indicate that this forest site is a stable enzootic focus.

### Mosquito Traps

Hamster-baited traps were used for detection of natural VEEV vectors. These traps were a version of the Trinidad No. 10 trap (*19*) with the following modifications: 1) the metal can comprising the trap opening was replaced by a polyvinyl chloride pipe, 9 cm in diameter; 2) the cylindrical animal cage was enlarged to 11 cm in diameter and 12 cm in height; 3) the roof was constructed from plexiglass; and 4) the opening for mosquito aspiration was a simple buttonhole sewn into the polyester collection net (Figure). The traps were baited with adult golden Syrian hamsters obtained from a colony maintained at the Instituto Nacional de Salud in Bogota. Baited traps were suspended approximately. 1.5 m above the ground and placed in transects at 10-m intervals. Carrots and rat chow were provided for food and water. The traps were checked each morning between 0600 and 0800 h, and some were also checked in the evening between 1700 and 1900 h. Mosquitoes were removed from the traps by using an aspirator, and the daily or semi-daily collections from each trap were frozen as a single pool in a plastic bottle immersed in liquid nitrogen vapor. When hamsters within the traps became moribund or died, serum samples were obtained by cardiac puncture or their hearts were dissected aseptically and frozen for virus isolation.

**Figure:**
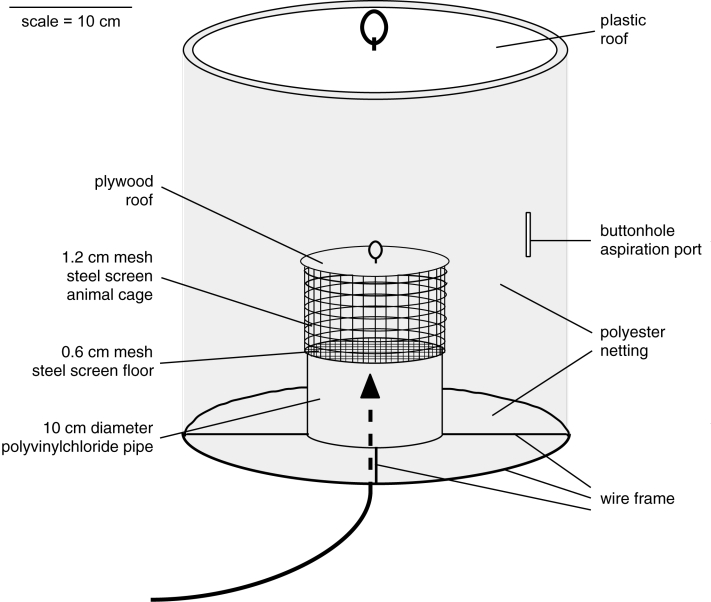
Major features of hamster-baited traps used to identify vectors of *Venezuelan equine encephalitis virus*. Arrow shows entry route of mosquitoes.

### Detection of Natural Transmission to Hamsters

To confirm VEEV infection in dead or moribund hamsters, virus was isolated from a 10% heart tissue suspension in Eagle’s minimal essential medium (MEM), supplemented with 20% fetal bovine serum (FBS) and antibiotics. The suspension was prepared in a Ten Broeck tissue grinder and centrifuged at 15,000 x *g* for 5 min; 200 μL of the supernatant was added to a 25-cm^2^ flask containing a monolayer of Vero cells and adsorbed for 1 h at 37°C; 6 mL of additional MEM containing 2% FBS was then added. Cultures were incubated at 37°C for 5 days or until cytopathic effects were evident.

Mosquito pools from traps in which hamster infection with VEEV was confirmed were assayed for infectious virus. Pools containing 1–40 individuals of each mosquito species were triturated with a Minibeadbeater (BioSpec Products, Inc., Bartlesville, OK) or a Ten Broeck tissue grinder containing 1.0 mL of MEM supplemented with 20% FBS, penicillin, streptomycin, and amphotericin B. The triturated pool was centrifuged for 5 min at 15,000 x *g*, and 200 µl of the supernatant was added to a 10-mL plastic tube or a 25-cm^2^ cell culture dish containing a monolayer of Vero cells and 2–5 mL of MEM. Cultures were monitored for cytopathic effects for 5 days.

### Genetic and Antigenic Characterization of VEEV Isolates

Viruses isolated from hamster heart tissue suspensions and mosquito pools were characterized antigenically by using immunofluorescence of infected cells and a panel of monoclonal antibodies described previously (*20*). Subtype ID VEEV isolates were further characterized genetically by reverse transcription–polymerase chain reaction (PCR) amplification of an 856-nucleotide portion of the PE2 (sometimes called p62) envelope glycoprotein precursor gene as described previously (*8*), followed by single-stranded conformation polymorphism (SSCP) or sequence analysis (*9*). For SSCP analysis, PCR products were purified on agarose gel by using the QIAquick Gel Extraction Kit (QIAGEN, Valencia, CA). A 2-μl volume of the PCR amplicon DNA suspension was mixed with 8 μl of SSCP loading buffer (95% formamide, 0.05% bromophenol blue, and 0.05% xylene cyanol). The DNA was heated to 95°C for 5 minutes, rapidly cooled on ice, loaded onto an 8% polyacrylamide gel, and underwent electrophoresis in 1X Tris-borate EDTA buffer at room temperature for 20 h at 8 mA. Single-stranded DNA products were visualized by using silver staining (*21*). SSCP patterns were compared by measuring the migration of single-stranded DNA of the various isolates in comparison to one another and to a standard DNA ladder.

## Results

For vector identification studies, 87 hamsters were exposed in traps within the Monte San Miguel Forest for 5–7 days. Of these, 38 became moribund or died and were processed for virus isolation. VEEV was isolated from 37 hamsters, and the mosquito collections from the corresponding traps were assayed for virus.

In 18 of the traps yielding infected hamsters, a vector species was identified by using the following criteria: 1) the hamster died at least 24 h after the collection of the presumed vector, consistent with the incubation time of VEEV in hamsters (*22*); 2) during the first day in which infected mosquitoes were collected from the trap, only one species pool had a high titer (>5.0 log_10_ PFU/pool) consistent with an infectious mosquito, as determined by previous experimental studies of enzootic VEEV vectors (*18*,*23*–*26*); 3) the remaining pools, from the first day in which infected mosquitoes were collected, were uninfected, or had low titers (<5.0 log) shown previously to be inconsistent with an infectious mosquito (*18*,*23*–*26*); 4) the mosquito collections on the days subsequent to that of the vector collection were mostly infected, reflecting hamster viremia and the ingestion of infectious blood by mosquitoes biting >12 h after the transmission event; and 5) virus isolates from the hamster and corresponding vector were indistinguishable antigenically and genetically with SSCP analysis, sequencing, or both. In 18/37 infected hamster events studied, these criteria were fulfilled, vector was identified unambiguously. Typical data for one of these transmission events (hamster 164) is shown in Table 1. In this example, transmission by *Cx. vomerifer* occurred < 24 h after exposure of the trap*,* and the vector pool had a titer of 5.8 log_10_ PFU/pool. The other two infected pools from day 2, *Cx. pedroi* and *Aedes serratus*, had log titers <3.3, indicating that they were not capable of transmission. These pools presumably contained one or more mosquitoes that engorged on the hamster after viremia began, probably just before the daily trap collection. On the next day, all mosquito pools contained infectious virus in their midguts, representing viremic hamster blood ingested by mosquitoes within the trap.

**Table 1:** Mosquito collections from hamster-baited trap no. 164

April 8, 1999			April 9, 1999			April 10, 1999		
Species	Fraction of pools positive	Pool titers	Species	Fraction of pools positive	Pool titers	Species	Fraction of pools positive	Pool titers
Culex (Melanoconion) pedroi	0/1	NT^a^	Cx. (Mel.) pedroi	1/1	3.3	Cx. (Mel.) pedroi	1/1	5.9
*Cx. (Mel.) spissipes*	0/1	NT	*Cx. (Mel.) spissipes*	0/1	NT	*Cx. (Mel.) spissipes*	1/1	4.7
*Cx. (Mel.) vomerifer*	0/1	NT	*Cx. (Mel.) crybda*	0/1	NT	*Cx. (Mel.) ferreri*	1/1	4.8
*Cx. (Mel.) adamesi*	0/1	NT	*Cx. (Mel.) vomerifer^b^*	1/1	5.8	*Cx. (Mel.) vomerifer*	1/1	5.5
*Aedes serratus*	0/1	NT	*Cx. (Mel.) adamesi*	0/1	NT	*Cx. (Mel.) adamesi*	1/1	5.1
*Cx. (Cx.) nigripalpus*	0/1	NT	*Ae. serratus*	1/1	<2	*Ae. serratus*	1/1	5.2
*Cx. (Ae.) amazonensis*	0/1	NT	*Cx. (Cx.) nigripalpus*	0/1	NT	*Cx. (Cx.) nigripalpus*	1/1	5.2
*Coquilletidia venezuelensis*	0/1	NT	*Cx. (Ae.) amazonensis*	0/1	NT	*Cx. (Ae.) amazonensis*	1/1	4.9
*Ae. fulvus*	0/1	NT	*Ae. fulvus*	0/1	NT	*Cx (Ae.) accelerans*	1/1	4.5
						*Mansonia titillans*	1/1	5.2

^a^NT, not tested.

^b^Incriminated vector pool.

A total of 18 transmission events were characterized as described above. The most common interval of collection of the identified vector was 24–48 h after exposure, reflecting a very high level of enzootic VEEV transmission in the Monte San Miguel Forest. *Cx. vomerifer* was implicated in 11 of these events, *Cx. pedroi* in 5, and *Cx. adamesi* in 2 transmissions (Table 2). The minimum infection/transmission rate for the mosquitoes we collected could not be determined directly because we did not identify the mosquito collections for traps where transmission to the hamster did not occur. However, rates on the order of 1/200–1/1000 can be estimated for these three vector species if the species composition is assumed to be similar in traps where transmission did not occur. Even if this assumption is incorrect, the error in this estimate should not be more than twofold because VEEV transmission occurred in most traps.

**Table 2:** Mosquito vector species identified in transmission of *Venezuelan equine encephalitis virus* to hamsters

Hamster no.	Vector species	Titer of vector pool^a^	Collection interval of vector pool (h)	Titer of other mosquito pools in the same collection as the vector^a^	Fraction of mosquito species infected on the subsequent day’s collection^a^
65	*Culex pedroi*	5.2	0–24	<2	6/10
66	*Cx. vomerifer*	5.0	48–72	<2	18/18
144	*Cx. vomerifer*	5.4	24–48	<2	NA
150	*Cx. adamesi*	5.5	24–48	<2	9/9
164	*Cx. vomerifer*	5.8	0–24	≤3.3	10/10
172^b^	*Cx. vomerifer*	5.4	24–48	≤3.8	9/9
184	*Cx. pedroi*	5.1	0–24	≤2.3	6/6
186	*Cx. vomerifer*	6.1	0–24	<2	9/9
264	*Cx. vomerifer*	5.4	0–24	>3.9	12/16
272	*Cx. pedroi*	5.1	0–24	<2	11/11
277	*Cx. adamesi*	5.3	0–24	<2	18/18
279	*Cx. vomerifer*	6.1	24–48	<2	14/16
286	*Cx. vomerifer*	5.5	120–144	<2	14/15
287	*Cx. pedroi*	5.4	0–24	<2	15/15
296	*Cx. vomerifer*	5.7	0–24	>4.9	10/10
290	*Cx. pedroi*	5.4	0–24	<2.8	13/15
304	*Cx. vomerifer*	5.3	48–72	<2	8/8
305	*Cx. vomerifer*	5.7	2	≤2.1	8/8

^a^Log_10_ Vero PFU per pool

^b^A second infected pool of *C. vomerifer* with a log titer of 3.8 was collected on day 1, but was presumed not to have been transmitted to the hamster due to its low titer.

## Discussion

Use of Hamster-Baited Traps for Arbovirus Vector Identification

Traditional criteria for arthropod vector identification include the following: 1) demonstration of feeding or other effective contact with pathogen’s host; 2) association in time and space of the vector and pathogen; 3) repeated demonstration of natural infection of the vector, and 4) experimental transmission of the pathogen by the vector (*27*). Infection rates for arbovirus vectors tend to be relatively low, usually <1%. Therefore, fulfillment of these criteria for arbovirus vectors usually relies on the capture of large numbers of arthropods for virus isolation, followed by experimental laboratory transmission studies to ensure that species found infected in nature are competent vectors. Although this strategy is the most comprehensive and unbiased, it is extremely costly and time consuming, accounting for the relative paucity of information on natural vectors of many arboviruses. Some studies of VEEV vectors have also relied on oral infection from experimentally infected hamsters with viremia levels of very high titer, on the order of 8 log_10_ PFU/mL (*28*,*29*), a titer at least 100–1,000 times greater than that generated by experimentally infected rodent reservoir hosts (*30*,*31*), equines (*13*,*30*,*32*), or naturally infected humans (*8*,*33*) (Some studies of equine viremia have yielded titers of >10^8^ suckling mouse intracerebral 50% lethal doses, but this method for quantifying VEEV titers is 100- to 1000-fold more sensitive than PFU [*30**,**34*]). Results from these studies are therefore inconclusive regarding natural transmission potential.

Other investigators have streamlined the vector identification process by collecting suspected vectors and sorting them according to species, then exposing single-species pools to naïve animals in a field or laboratory setting to detect transmission (*16*,*18*). We have taken this approach one step further by combining collection and transmission detection using hamster-baited traps. This method simplifies the vector identification process in several ways: 1) Hamster-baited traps attract and capture only arthropod species that are attracted to small mammals, the natural reservoir hosts of the enzootic VEEV (*Proechimys* spp. spiny rats in the case of subtype ID VEEV circulating in this focus [*35*]), minimizing collection and mosquito processing efforts. 2) Arthropod collections from traps where no transmission occurs do not need to be sorted, greatly reducing a laborious step in the vector identification process. 3) Only a small number of arthropod pools must be tested for virus, eliminating much of the cost, labor, and biosafety hazard associated with traditional vector identification approaches. In addition, the hamster-baited traps can serve as sentinels for detection of active virus circulation in a forest and reveal the presence of other viruses in a focus. However, unlike other sentinel enclosures that allow arthropods to escape after biting a viremic bait animal and thereby initiate artificial amplification, the hamster-baited traps capture most of the arthropods that bite the viremic host and prevent most or all artificial amplification. A similar strategy for detecting transmission of western equine encephalitis and St. Louis encephalitis viruses to chickens in baited traps was described by Reeves et al. (*36*).

Using these hamster-baited traps alone, we were not able to measure directly the capture efficiency of our traps. However, in the case of five infected hamsters, the lack of any collections with a single or few high titer mosquito species pools on the day preceding total infection of collected mosquitoes indicates that the arthropod responsible for transmission may have escaped. In other cases, two or more mosquito pools collected on the first day virus was detected had titers consistent with infectious vectors, precluding vector identification. We are currently experimenting with funnel-shaped openings to reduce the frequency of vector escape from this trap design. As with any passive trap design, a compromise between ease of vector entry and frequency of escape must be sought to maximize collections.

### Enzootic Vectors of Venezuelan Equine Encephalitis Complex Viruses

Previous studies of VEE complex enzootic transmission have each identified a single, principal mosquito species in a given geographic region. All of these species, including *Cx.* *portesi* (*14*), *Cx. cedecei* (*15*), *Cx. aikenii* *sensu lato* (*ocossa* and *panocossa*) (*16*,*17*), and *Cx.* *taeniopus* (*18*) are members of the Spissipes section of the subgenus *Melanoconion* within the genus *Culex* (*37*). Previous studies of enzootic VEEV transmission in the Catatumbo region of northeastern Colombia also suggested that *Cx. pedroi* might be the principal vector, based on abundance in active foci (*38*). *Cx. vomerifer* from Iquitos, Peru, also has been shown to be susceptible to infection by several strains of VEEV (*28*), but was only tested after mosquitoes ingested 8 log_10_ PFU/mL from viremic hamsters, a viremia titer at least 100 times greater than that generated by experimentally infected rodent reservoir hosts (*30*,*31*). Our findings of at least three enzootic vectors of subtype ID VEEV in Colombia contrast with the findings of all previous studies of enzootic VEEV vectors, which suggested that enzootic VEEV strains are each adapted to a single, principal vector species (*13*,*18*,*39*–*41*). In Colombia, subtype ID VEEV appears to utilize efficiently both *Cx. vomerifer* and *Cx. pedroi* in the Magdalena Valley. *Cx. adamesi*, which is usually less abundant in the Monte San Miguel Forest, appears to serve as a secondary vector.

All three of the mosquito species that we identified as VEEV vectors are members of the Spissipes section of the subgenus *Culex* (*Melanoconion*), bringing the total to seven confirmed vectors within this section of closely related mosquitoes. The genetic or ecologic basis for the exclusive use of these mosquitoes by enzootic VEE complex viruses deserves further study. Hypotheses to explain this phenomenon include possible shared, derived characteristics of the Spissipes section, such as particularly high susceptibility to infection by enzootic VEE complex viruses, a particularly high degree of association with the *Proechimys* spp (*35*). and other small mammalian reservoir hosts (*13*), or both. Mosquito longevity and population sizes in habitats that support large populations of reservoir hosts may also favor transmission by members of the Spissipes section (*35*).

### Role of Enzootic Vectors in VEEV Emergence and Disappearance

Identification of the principal enzootic vectors (*Cx. vomerifer* and *Cx. pedroi)* of subtype ID VEEV strains believed to be closely related to epizootic progenitors will allow us to assess the role of these mosquitoes in the generation of mutations that mediate VEE emergence by enhancing equine viremia and infection of epizootic mosquito vectors such as *Ae. taeniorhynchus*. The hypothesis that epizootic VEEV is not recovered from sylvatic foci because these strains lose their fitness for the enzootic vectors (*25*) can also be tested in the two principal vectors that we identified.
